# NLRP3 Inflammasome Activation Controls Vascular Smooth Muscle Cells Phenotypic Switch in Atherosclerosis

**DOI:** 10.3390/ijms23010340

**Published:** 2021-12-29

**Authors:** Fabienne Burger, Daniela Baptista, Aline Roth, Rafaela Fernandes da Silva, Fabrizio Montecucco, François Mach, Karim J. Brandt, Kapka Miteva

**Affiliations:** 1 Division of Cardiology, Foundation for Medical Research, Department of Medicine, Specialized Medicine, Faculty of Medicine, University of Geneva, Av. de la Roseraie 64, 1211 Geneva, Switzerland; Fabienne.Burger@unige.ch (F.B.); Daniela.Baptista@unige.ch (D.B.); Aline.Roth@unige.ch (A.R.); rfdasilva.ufmg@gmail.com (R.F.d.S.); Francois.Mach@hcuge.ch (F.M.); Karim.Brandt@hcuge.ch (K.J.B.); 2 Department of Physiology and Biophysics, Institute of Biological Sciences, Federal University of Minas Gerais, Belo Horizonte 6627, Brazil; 3 Swiss Institute for Translational and Entrepreneurial Medicine, Freiburgstrasse 3, 3010 Bern, Switzerland; 4 Ospedale Policlinico San Martino Genoa—Italian Cardiovascular Network, 10 Largo Benzi, 16132 Genoa, Italy; fabrizio.montecucco@unige.it; 5 First Clinic of Internal Medicine, Department of Internal Medicine and Centre of Excellence for Biomedical Research (CEBR), University of Genoa, 6 Viale Benedetto XV, 16132 Genoa, Italy

**Keywords:** NLRP3 inflammasome activation, vascular smooth muscle, vascular smooth muscle phenotypic switch, atherosclerosis, atherosclerosis plaques stability

## Abstract

(1) Background: Monocytes and nucleotide-binding oligomerization domain-like receptor protein 3 (NLRP3) inflammasome orchestrate lipid-driven amplification of vascular inflammation promoting the disruption of the fibrous cap. The components of the NLRP3 inflammasome are expressed in macrophages and foam cells within human carotid atherosclerotic plaques and VSMCs in hypertension. Whether monocytes and NLRP3 inflammasome activation are direct triggers of VSMC phenotypic switch and plaque disruption need to be investigated. (2) Methods: The direct effect of oxLDL-activated monocytes in VSMCs co-cultured system was demonstrated via flow cytometry, qPCR, ELISA, caspase 1, and pyroptosis assay. Aortic roots of VSMCs lineage tracing mice fed normal or high cholesterol diet and human atherosclerotic plaques were used for immunofluorescence quantification of NLRP3 inflammasome activation/VSMCs phenotypic switch. (3) Results: OxLDL-activated monocytes reduced α-SMA, SM22α, *Oct-4*, and upregulation of *KLF-4* and macrophage markers MAC2, F4/80 and CD68 expression as well as caspase 1 activation, IL-1β secretion, and pyroptosis in VSMCs. Increased caspase 1 and IL-1β in phenotypically modified VSMCs was detected in the aortic roots of VSMCs lineage tracing mice fed high cholesterol diet and in human atherosclerotic plaques from carotid artery disease patients who experienced a stroke. (4) Conclusions: Taken together, these results provide evidence that monocyte promote VSMC phenotypic switch through VSMC NLRP3 inflammasome activation with a likely detrimental role in atherosclerotic plaque stability in human atherosclerosis.

## 1. Introduction

Cardiovascular diseases (CVD) are still the predominant cause of death and morbidity, with atherosclerosis as the main underlying cause [1]. Atherosclerosis is a lipid-driven, chronic inflammatory disease characterized by the build-up of subendothelial deposition of cholesterol and the formation of leukocyte-rich plaques in the intimal layer of the arteries. Inflammation plays a major role in promoting the disruption of the fibrous cap that covers the atherosclerotic plaque, resulting in myocardial infarction and stroke [2]. The fibrous cap is composed mainly of VSMCs. Expansion of monocytes is an independent risk factor for CVD, causally linked to the enlargement of the atherosclerotic lesion [3]. Oxidized low-density lipoprotein (oxLDL)-activated monocytes enhance atherogenesis by triggering inflammatory cascades and overproduction of reactive oxygen species (ROS), and the accumulation of monocyte-derived macrophages [3]. The uptake of oxLDL by macrophages results in the formation of lipid-laden foam cells with impaired migratory ability, that dies and forms a necrotic core that further contributes to destabilizing the plaques [4,5,6]. Nucleotide-binding oligomerization domain-like receptor protein 3 (NLRP3) inflammasome activation has been shown to be a powerful mediator of inflammatory response via the release of the pro-inflammatory mediators interleukin-1β (IL) and IL-18 that boost lipid deposition, foam cell accumulation, and atherosclerosis progression [7]. Furthermore, the CANTOS trial confirmed the inflammatory hypothesis of atherosclerosis as well as the significant role of IL-1β in the pathogenesis of atherosclerosis, although this did not result in approval of the studied IL-1β-inhibitor canakinumab due to higher rates of infection in the active treatment group [8]. Interestingly, 60% to 70% of foam cells in atherosclerotic lesions are of VSMC, not leukocyte origin, but whether NLRP3 inflammasome activation plays a role in VSMC phenotypic switch is not known. IL-1β is a proinflammatory cytokine exerting its functions through autocrine, paracrine, or endocrine mechanisms [9]. Moreover, IL-1β has been shown to induce its own gene expression in various cell types in an amplification loop manner called autoinduction [10,11]. IL-1β promotes endothelial dysfunction [12], leukocyte-endothelial cell adhesion, procoagulant activity, and recruitment of leukocytes [12] and neutrophils promoting atherogenesis and plaque rupture [13,14]. Interestingly, it has been shown that IL-1β triggers proliferation, IL-6 and platelet-derived growth factor production in VSMCs [10]. A recent publication by the group of Owens demonstrated that after using VSMC Il1r1 knockout mice, IL-1 signaling is required for the investment of VSMCs into the fibrous cap in a model of advanced atherosclerosis [15]. However, the effects of an IL-1β-neutralizing antibody deleterious to fibrous cap stability in mice [15] proved to be beneficial in reducing cardiovascular events in the CANTOS trial in humans [8]. Since NLRP3 inflammasome activation was shown to be an important mechanism driving atherogenesis, inflammation, and foam cell formation, it could emerge also as a crucial mechanism triggering VSMC phenotypic switch and subsequently plaque destabilization. Until now, this hypothesis has not been investigated and it could open a door to the revelation of a new mechanism in vascular pathology.

## 2. Results

### 2.1. OxLDL-Activated Monocytes Promote VSMC Phenotypic Switch

**Figure 1:**
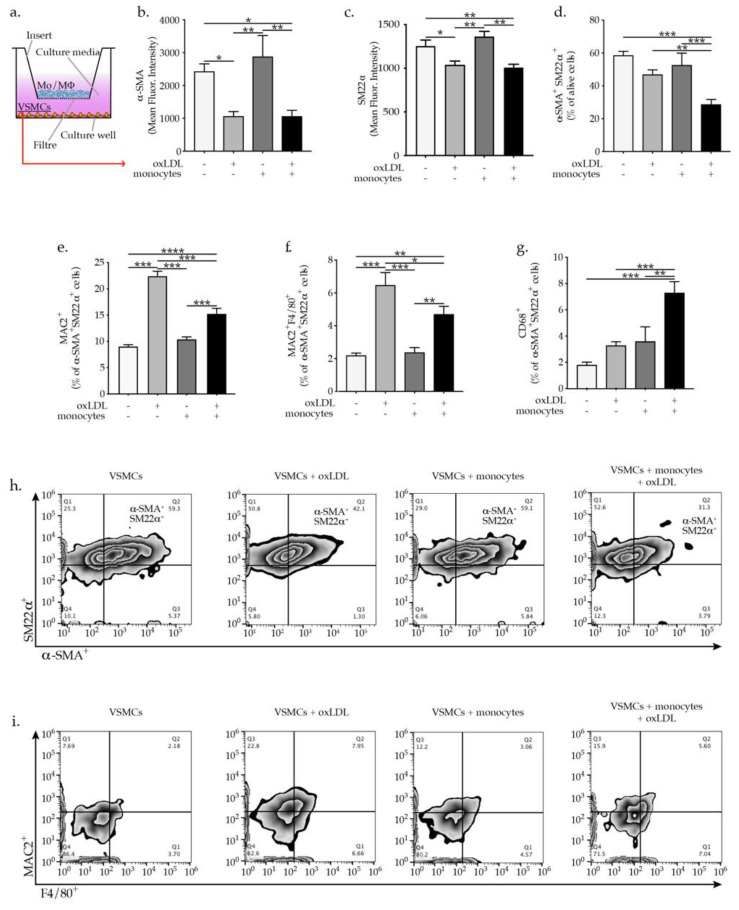
(**a**) Scheme of in vitro experimental setting and graph bars represent the mean ± SEM of flow cytometry analysis of VSMCs phenotypic switch (**b**) α-SMA^+^, (**c**) SM22α^+^ expressed as mean fluorescence intensity and (**d**) α-SMA^+^SM22α^+^, (**e**) MAC2^+^, (**f**) MAC2^+^F4/80^+^, and (**g**) CD68^+^ cells, expressed as percentage of indicated VSMC cells upon oxLDL treatment or co-culture with monocytes, with n = 6/group and * *p*  <  0.05, ** *p*  <  0.01, *** *p*  <  0.001, and **** *p*  <  0.0001, one-way ANOVA. Representative flow cytometry zebra plots of (**h**) α-SMA^+^SM22α^+^ and (**i**) MAC2^+^F4/80^+^ expression quantification.

**Figure 2:**
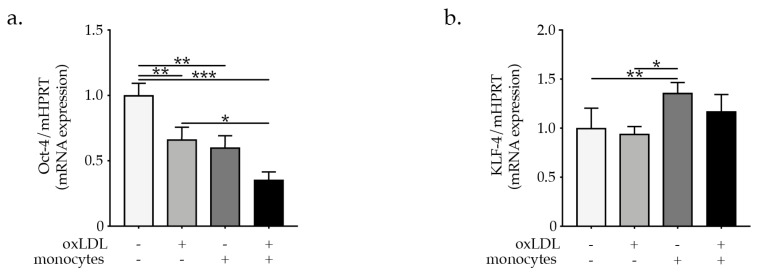
Graph bars represent the mean ± SEM of mRNA expression of (**a**) *Oct-4* and (**b**) *KLF-4* indicating VSMC phenotypic switch upon oxLDL treatment or co-culture with monocytes or or oxLDL-activated monocytes, as indicated, with n = 6/group and * *p* < 0.05, ** *p* < 0.01, and *** *p* < 0.001, one-way ANOVA.

VSMCs were isolated from the aortic arch of 8 to 12 weeks old C57BL/6 mice and after VSMCs expansion, the phenotype and purity were confirmed by staining with anti-mouse α-SMA, SM22α, and CD31 (endothelial cell marker) and CD90 (fibroblasts cell marker). Supplementary Figure S1a shows that the obtained VSMCs expressed the VSMC-specific markers α-SMA, SM22α, but are negative for the endothelial cells marker (CD31) as well as the fibroblasts marker (CD90). Mouse monocytes were isolated from bone marrow of C57BL/6 mice and after purity check up (Supplementary Figure S1b) were used in co-culture experiments with VSMCs. OxLDL-activated monocytes are known to trigger inflammatory cascades, promoting endothelial dysfunction and enhancing atherogenesis [4,5,6]. To demonstrate the effect of oxLDL on monocytes activation we showed a dose-dependent ROS production and IL-6 expression in oxLDL-activated monocytes as indicated by the increase of the mean fluorescent intensity of carboxylated H2DCFDA and upregulated expression of IL-6 (Supplementary Figure S1c,d). To study the direct effect of monocytes and particularly the role of oxLDL-activated monocytes on VSMCs phenotypic modulation, we performed co-culture experiments in a trans-well system in which VSMCs were plated in the plate wells while monocytes or oxLDL-activated monocytes were added to the well cell culture inserts (Figure 1a). Monocytes oxLDL activation was induced upon direct supplementation of oxLDL to well cell culture inserts impermeable to oxLDL to ensure monocytes restricted activation. Treatment of VSMCs with oxLDL resulted in a pronounced reduction in the expression of the VSMC-specific markers α-SMA and SM22α. Importantly, the supplementation of VSMCs with oxLDL-activated monocytes resulted in a pronounced reduction in the expression of VSMC-specific markers α-SMA and SM22α expressed as mean fluorescence intensity (Figure 1b,c). Interestingly, the percentage of double positive α-SMA^+^SM22α^+^ VSMCs was only prominently downregulated in VSMC upon co-cultured with oxLDL-activated monocytes (Figure 1d). As expected, oxLDL treatment of VSMCs promoted increased expression of macrophages markers MAC2 and F4/80 in VSMCs (Figure 1d,e). Furthermore, co-culture with oxLDL-activated monocytes elevated the expression of MAC2 and F4/80 (Figure 1d,e) in VSMCs as well as the expression of CD68 which was only significantly elevated post co-culture with oxLDL-activated monocytes (Figure 1f). In line with our hypothesis, monocytes and oxLDL-activated monocytes downregulated the expression of the transcription factor *Oct-4* in VSMC, known to be important in preserving VSMC contractile phenotype [16], while *KLF-4* showed to promote VSMCs phenotypic modulation [17,18], was upregulated in VSMCs (Figure 2a,b). Taken together, these results demonstrate that oxLDL activated monocytes are effective at promoting VSMCs phenotypic switch and their transdifferentiation to macrophages-like cells.

### 2.2. Monocytes Promote VSMC NLRP3 Inflammasome Activation

**Figure 3:**
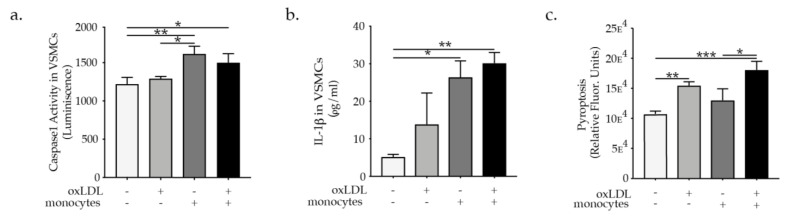
Graph’s bar represent the mean ± SEM of (**a**) caspase 1 activation, (**b**) IL-1β secretion, and (**c**) pyroptosis in VSMC upon oxLDL treatment or monocytes or oxLDL-activated monocytes supplementation as indicated, with n  =  6/group and * *p*  <  0.05, ** *p*  <  0.01, and *** *p*  <  0.001, one-way ANOVA.

**Figure 4:**
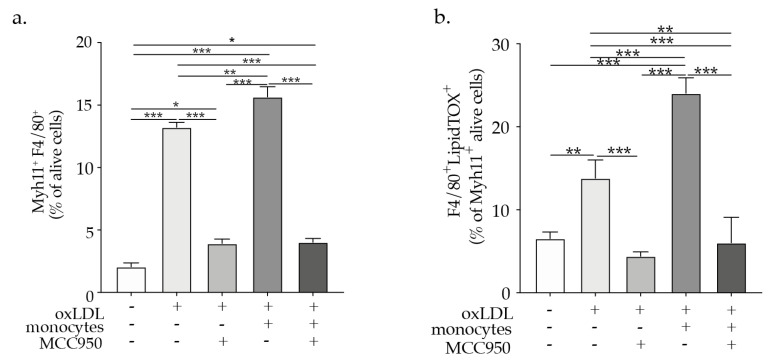
Bars graphs represent the mean ± SEM of (**a**) Myh11^+^F4/80^+^-expressing VSMCs and (**b**) F4/80^+^LipidTOX^+^ (foam cells) formation as a percentage of alive cells as indicated upon oxLDL or monocytes, or oxLDL-activated monocytes supplementation in the presence or absence of MCC950, with n = 6/group and * *p* < 0.05, ** *p* < 0.01, *** *p* < 0.001, one-way ANOVA.

Despite a great deal of evidence pointing out the critical role of monocytes/macrophages in atherosclerosis vascular diseases [19], previous studies have not clearly defined the inflammatory effect of monocytes on VSMCs in atherosclerosis. Furthermore, NLRP3 inflammasome activation was shown to be an important mechanism driving atherogenesis, inflammation, and foam cells formation, therefore it could emerge as a crucial mechanism triggering VSMCs phenotypic switch. However, until now this hypothesis has not been investigated and it could open a door to the revelation of a new mechanism in vascular pathology. To demonstrate the effect of monocytes and oxLDL on VSMCs NLRP3 inflammasome activation we performed co-culture experiments where the direct effect of monocytes or oxLDL-activated monocytes on VSMCs NLRP3 inflammasome activation was investigated. We used a trans-well system in the co-culture experiments in which VSMCs were plated in the plate wells while monocytes or oxLDL-activated monocytes were added to oxLDL impermeable trans well inserts, as previously described. In order to facilitate inflammasome assembly, NLRP3 interacts with the N-terminus of the adapter protein ASC via PYD–PYD interactions; the C-terminus of ASC has a caspase recruitment domain (CARD) that binds to procaspase-1 via CARD–CARD interactions triggering caspase dimerization and subsequent activation. Interestingly due to its prion-like properties ASC forms large fibrillar aggregates known as “specks” [20]. Using the above-described co-culture system we could demonstrate that monocytes, as well as oxLDL-activated monocytes, promote ASC specks formation as visualized by confocal microscopic analysis of ASC speck formation in VSMCs (Supplementary Figure S2a). To further confirm that ASC speck formation results in the activation of caspase-1 involved in the maturation of IL-1β into a biologically active form, and cleavage of gasdermin D (GSDMD) to promote pyroptotic cell death (pyroptosis) [21], we investigated caspase 1 activation and pyroptosis in VSMCs treated with oxLDL or co-cultured with monocytes or oxLDL activated monocytes as described previously. Caspase 1 activity in VSMCs was raised particularly when VSMCs were exposed to paracrine mediators from monocytes as well as oxLDL-activated monocytes in the co-culture system (Figure 3a). To measure IL-1β secretion specifically in VSMCs the trans-well inserts were removed and VSMC were supplemented with a fresh medium to be able to evaluate IL-1β secretion specifically by VSMCs. In line with the caspase 1 activation induction, the co-culture with monocytes or oxLDL activated monocytes triggered IL-1β secretion by VSMCs (Figure 3b). Pyroptosis programmed cell death associated with NLRP3 inflammasome activation [21] was more pronouncedly induced in VSMCs after they were exposed to paracrine factors release by oxLDL-activated monocytes in the co-culture system (Figure 3c). In parallel, VSMCs treated with oxLDL or co-cultured with monocytes or oxLDL-activated monocytes showed a pronounced increase in cell death as evaluated by VSMCs positive staining for the red live/dead propidium iodide and 7-AAD staining apoptotic cells and quantified by flow cytometry analysis (Supplementary Figure S1e). Under hypercholesteremia, Apoe^−/−^ mice exhibit an increased percentage of VSMCs undergoing phenotypic switch and expressing NLRP3 as indicated by co-expression of α-SMA, CD68, and NLRP3 (Supplementary Figure S2b). Moreover, NLRP3 inhibitor MCC950 abrogated oxLDL or oxLDL-activated monocytes-induced VSMC phenotypic switch as evident by the pronounced reduction in F4/80 expression in Myh11 positive VSMCs and VSMCs foam cells (F4/80^+^ LipidTOX^+^) (Figure 4a,b). These results provide evidence for the involvement of NLRP3 in VSMCs phenotypical switch upon hypercholesteremia or in the presence of oxLDL-activated monocytes.

### 2.3. IL-1β Promotes VSMC Phenotypic Switch and Transdifferentiation to Macrophages-Like Cells

**Figure 5:**
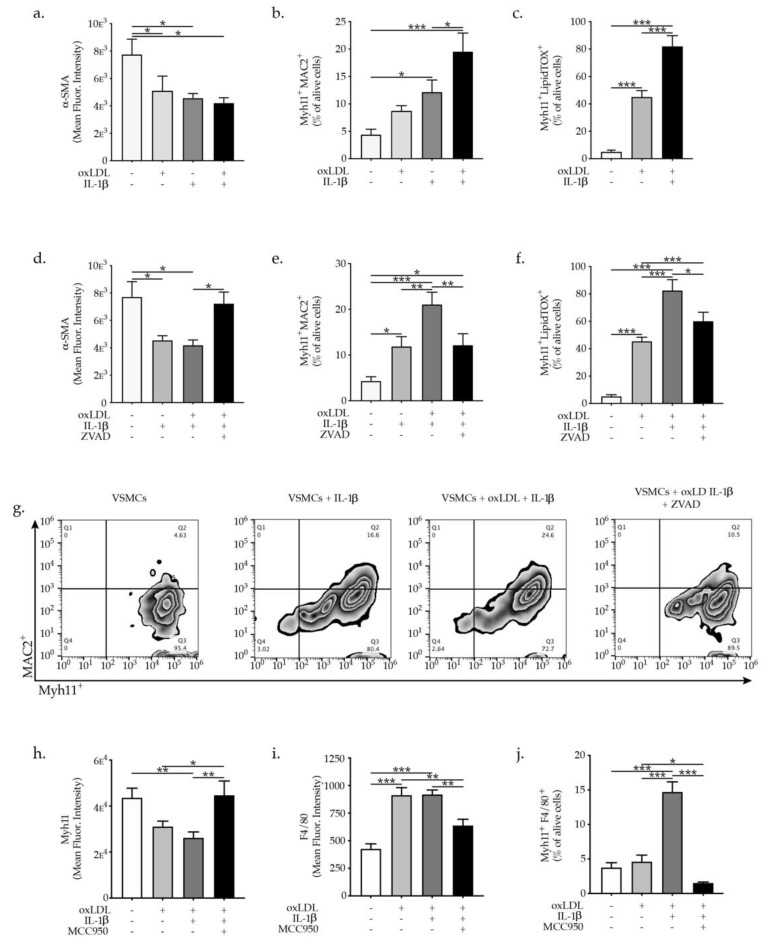
Graph bars represent the mean ± SEM of (**a**) α-SMA^+^, (**b**) Myh11^+^MAC2^+^ expression and (**c**) Myh11^+^LipidTOX^+^ (foam cells) formation of VSMCs upon oxLDL and IL-β treatment as indicated, with n  =  6/group and * *p*  < 0.05, ** *p* < 0.01, *** *p*  <  0.001. Graph bars represent the mean ± SEM of (**d**) α-SMA^+^, (**e**) Myh11^+^MAC2^+^, expressed as mean fluorescence intensity and (**f**) Myh11^+^LipidTOX^+^ (foam cells) expressed as a percentage of a alive VSMC upon oxLDL and/or IL-β and/or ZVAD treatment as indicated, with n  =  6/group and * *p*  <  0.05, ** *p*  <  0.01, *** *p*  <  0.001, one-way ANOVA. (**g**) Representative flow cytometry zebra plots of Myh11^+^MAC2^+^ cells. Graph bars represent the mean ± SEM of (**h**) Myh11^+^, (**i**) F4/80^+^ expressed as mean fluorescence intensity and (**j**) Myh11^+^F4/80^+^ as a percentage of alive VSMC upon oxLDL and/or IL-β and/or MCC950 treatment as indicated, with n  =  6/group and * *p*  <  0.05, ** *p*  <  0.01, *** *p*  <  0.001, one-way ANOVA.

To investigate whether IL-1β has a direct effect on promoting VSMCs phenotypic switch, we supplemented IL-1β to VSMCs. Treatment of VSMCs with 10 ng/mL of IL-1β for 7 days promoted pronounced reduction in the expression of α-SMA (Figure 5a). Furthermore, IL-1β treatment of VSMCs promoted a pronounced increase in the expression of macrophages markers MAC2^+^ (LGALS3^+^) while the combination of IL-1β in addition to oxLDL treatment further upraised the expression of MAC2 in Myh11^+^ VSMC cells (Figure 5b). Remarkably, IL-1β treatment resulted in a profound increase of lipids accumulation in VSMCs as evidenced by amplification of the Myh11 cells expressing LipidTOX as an indicator of lipids cell accumulation and subsequently foam cells formation (Figure 5c). These data not only support a critical role for IL-1β in induction of VSMCs phenotypic switch to macrophages-like cell but also reveal the involvement of IL-1β in VSMCs foam cells formation, with a critical role in atherosclerotic plaque stability. Interestingly, in the presence of ZVAD-FMK a cell-permeable pan-caspase inhibitor, the oxLDL and IL-1β-induced VSMC phenotypic switch were partly abrogated, as evident by a restoration of α-SMA expression in VSMCs as well as reduction of MAC2 expression in Myh11^+^ VSMCs (Figure 5d,e,g). Furthermore, ZVAD-FMK significantly diminished the percentage of Myh11 cells expressing LipidTOX (foam cells formation) in comparison to oxLDL and IL-1β-treated VSMCs (Figure 5f). These findings clearly demonstrate that inhibition of IL-1β signal transduction might be a way to regulate VSMCs phenotypic switch and foam cells formation. The specific involvement of NLRP3 inflammasome activation in VSMC phenotypic switch induced by oxLDL and IL-1β was demonstrated using MCC950 which is a potent highly specific small molecule inhibitor of both canonical and noncanonical activation of NLRP3 inflammasome leading to reduction of IL-1β production [22]. OxLDL and IL-1β promoted reduction in the expression of VSMCs-specific contractile protein Myh11^+^ while MCC950 supplementation completely restored the Myh11 expression in VSMCs (Figure 5h). Moreover, MCC950 reduced prominently the expression of the macrophage markers in VSMCs treated with oxLDL and IL-1β (Figure 5i,j). The presented findings clearly demonstrate the specific involvement of NLRP3 inflammasome activation in VSMC phenotypic switch since small molecule inhibitor of NLRP3 inflammasome MCC950 abrogated VSMCs phenotypic switch.

### 2.4. NLRP3 Inflammasome Inhibition Abrogates VSMCs Phenotypic Switch

**Figure 6:**
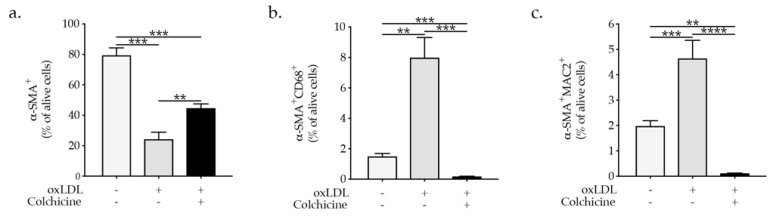
Graph bars represent the mean ± SEM of (**a**) α-SMA^+^, (**b**) α-SMA^+^CD68^+^, and (**c**) α-SMA^+^MAC2^+^ expression in VSMCs upon colchicine and/or oxLDL treatment as indicated, with n  =  5–6/group and ** *p*  <  0.01, *** *p*  <  0.001, **** *p*  <  0.0001 one-way ANOVA.

COLCOT (Colchicine Cardiovascular Outcomes Trial) and LoDoCo2 (Low Dose Colchicine2) trial demonstrated that low-dose colchicine is efficient in preventing major adverse cardiovascular events [23,24]. However, the precise mechanism of colchicine-mediated effects is not revealed. In this regard, we could demonstrate that oxLDL promoted VSMCs phenotypic switch, as indicated by the reduction in α-SMA expression and increased expression of CD68 and MAC2 in VSMCs, while colchicine treatment abrogated the VSMC phenotypic switch (Figure 6a–c). The presented data strongly suggest that hypercholesteremia induces NLRP3 inflammasome activation in VSMCs and subsequent VSMC phenotypic switch, and demonstrate the potential inhibitory effect of colchicine on oxLDL-induced VSMC phenotypic switch, which could potentially result in the prevention of plaque progression and destabilization.

### 2.5. Hypercholesteremia In Vivo Promotes NLRP3 Inflammasome Activation in VSMCs Associated with VSMCs Phenotypic Switch

**Figure 7:**
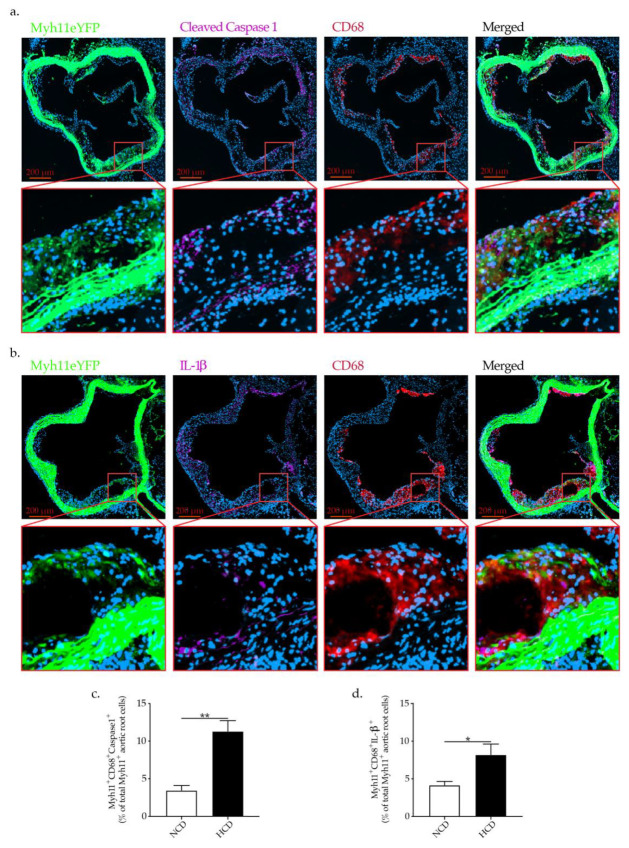
Representative immunofluorescence staining of Myh11eYFP^+^ cells, expressing (**a**) cleaved caspase 1, CD68 and (**b**) IL-1β, CD68 in the aortic roots of Apoe^−/−^ Myh11ERT2-CreR26R-eYFP mice showing NLRP3 inflammasome activation in VSMC undergoing phenotypic switch in vivo, taken by confocal microscopy (LSM 800 Airyscan). Graph bars show the mean ± SEM of (**c**) Myh11eYFP^+^ CD68^+^ Cleaved Caspase1^+^ and (**d**). Myh11eYFP^+^ CD68^+^ IL-1β co-expressing cells as a percentage of all Myh11eYFP^+^ cells in the aortic roots plaquesm with n = 8/group and * *p*  <  0.05, ** *p*  <  0.01, unpaired *t*-test.

To demonstrate that NLRP3 inflammasome activation in VSMCs is a relevant mechanism involved in VSMCs phenotypic switch in vivo, we used VSMCs lineage tracking mice. Apoe^−/−^ Myh11ERT2-CreR26R-eYFP mice with a tamoxifen-inducible recombinase driven by a VSMC-specific gene (Myh11) promoter in combination with reporter protein to facilitate specific labeling of VSMC in Apoe^−/−^ mice [25] were fed NCD or HCD. HCD-fed Apoe^−/−^ Myh11ERT2-CreR26R-eYFP mice showed significantly elevated levels of cholesterol and LDL-C as well as larger atherosclerotic lesions in the aortic roots as well as abdominal aorta in comparison to NCD-fed Apoe^−/−^ Myh11ERT2-CreR26R-eYFP mice (data not shown). Apoe^−/−^ Myh11ERT2-CreR26R-eYFP mice are an excellent model for the objective since they allow stable labeling of VSMCs at baseline, which facilitates VSMCs precise tracing and importantly the tracking of VSMC-derived cells during atherogenesis, even when VSMC characteristics might otherwise have been lost. Importantly, Apoe^−/−^ Myh11ERT2-CreR26R-eYFP mice exhibited pronounced NLRP3 inflammasome activation as demonstrated by cleaved caspase 1 and IL-1β expression in VSMCs (Myh11eYFP^+^ cells) undergoing phenotypic switch (co-expressing CD68) in the aortic roots (Figure 7a,b). Moreover, hypercholesteremia significantly increases the expression of cleaved caspase 1 and IL-1β in Myh11eYFP^+^ cell co-expressing CD68 in comparison to the mice fed NCD. (Figure 7c,d). These findings clearly demonstrate that that inflammasome activation is indeed involved in VSMC phenotypic switch in response to hypercholesteremia in vivo.

### 2.6. NLRP3-Inflammasome Activation in VSMCs Is Associated with Plaque Rupture in Human Carotid Artery Disease

**Figure 8:**
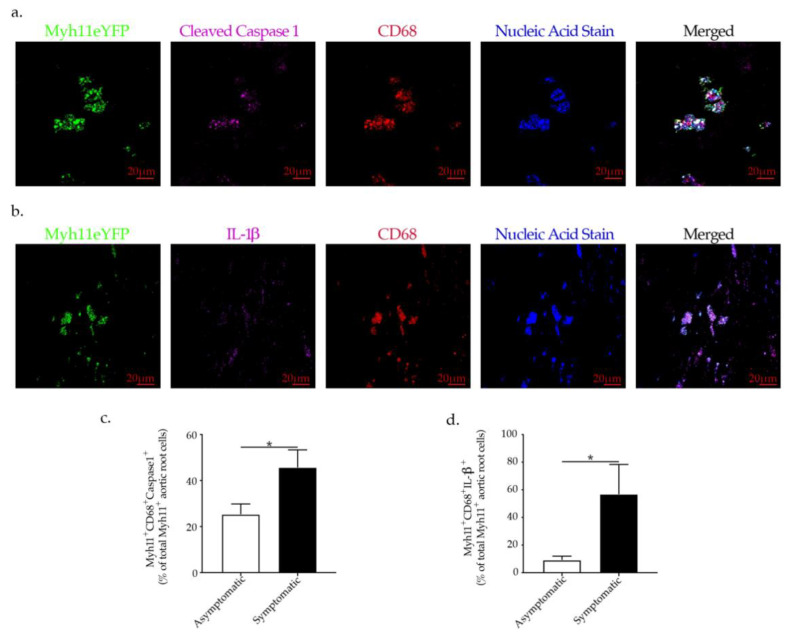
Representative immunofluorescence staining of Myh11^+^ cells, expressing (**a**) cleaved caspase 1, CD68 and (**b**) IL-1β, CD68 in human atherosclerotic plaques associated with NLRP3-inflammasome activation in VSMC and linked to plaque rupture in human carotid artery disease shown by confocal microscopy. Graph bars show the mean ± SEM of (**c**) Myh11^+^ CD68^+^ cleaved caspase 1 ^+^ and (**d**) Myh11^+^ CD68^+^ IL-1β^+^ co-expressing cells as a percentage of plaque Myh11 cells^+^ cells with n  =  12/group and * *p*  <  0.05, unpaired *t*-test.

To gain insight into a possible role of NLRP3 inflammasome activation in VSMCs phenotypic switch and its relevance for the destabilization of human atherosclerotic plaques, we performed immunofluorescence staining of human carotid atherosclerotic plaques derived from carotid artery disease patients. We found that VSMCs (Myh11^+^) undergoing transdifferentiation to macrophages-like cells in human atherosclerotic plaques co-express cleaved caspase 1 as well as IL-1β, indicating the involvement of NLRP3 inflammasome activation in VSMCs phenotypic switch in human atherosclerosis (Figure 8a,b). Furthermore, symptomatic patients who had experienced an ipsilateral ischemic stroke in comparison to asymptomatic patients (no ischemic events) had a significant increase in the number of Myh11^+^ Cleaved Caspase 1^+^ CD68^+^ as a percentage of the total Myh11^+^ present in human carotid atherosclerotic plaques in comparison to asymptomatic patients (Figure 8c). In line with the observed cleaved caspase 1 upregulation, we found a higher percentage of Myh11 ^+^CD68^+^ IL-1β^+^ cells present in human carotid atherosclerotic plaques of symptomatic patients versus asymptomatic CAD patients (Figure 8d). These findings imply that the increased number of VSMCs undergoing switch could have a causal role in human atherosclerotic plaque destabilization. Taken all together our data imply the involvement of NLRP3 inflammasome activation in VSMCs phenotypic switch with possible implications in human atherosclerotic plaque destabilization.

## 3. Discussion

Our present study provides evidence for the involvement of monocytes in triggering NLRP3 inflammasome signaling promoting VSMCs phenotypic switch and atherosclerosis progression. NLRP3 inflammasome activation and IL-1β signaling appeared to play a direct role in VSMCs phenotypic modulation. Our results provide insight into the direct role of monocytes and hypercholesteremia in triggering NLRP3 inflammasome activation involved in VSMCs phenotypic switch/foam cells formation with a possible implications for the destabilization of human atherosclerotic plaques.

Upon entrance to the intima, monocytes uptake ox-LDL and undergo macrophages foam cells formation via metabolization of ox-LDL through membrane scavenger receptors [26]. The accumulated foam cells are commonly found in early atherosclerotic lesions which can impact the functionality of VSMCs. Indeed, the interaction of VSMCs with monocytes/macrophages has been shown to promote matrix metalloproteinases production involved in VSMCs migration [27,28], affect the VSMCs phenotype and proliferative capacity [29,30,31], and promote VSMC apoptosis via Fas receptor-ligand binding to macrophages [32,33]. The present study reveals a major novel mechanistic controlling the initiation of VSMCs phenotypic switch/foam cell formation in atherosclerosis. Monocyte promotes VSMCs phenotypic switch to macrophages-like cells via VSMCs NLRPs inflammasome activation. We observed that monocytes and hyperlipidemia modulate VSMCs as follow: (1) Promote their phenotypic switch to macrophages-like cells; (2) reduce the expression of the transcription factor Oct-4 in VSMC, known to be important in preserving VSMC contractile phenotype [16], and upregulate KLF-4 expression shown to promote VSMC phenotypic modulation [17,18]; (3) trigger NLRP3 inflammasome activation and IL-1β secretion by VSMCs as well as; (4) induction of pyroptosis programmed cell death associated with NLRP3 inflammasome activation [21] and with the atherosclerosis plaque rupture [34]. Taking all together, monocytes and hypercholesteremia trigger VSMC phenotypic modulation, cholesterol accumulation, inflammasome activation, secretion of highly pro-inflammatory cytokine—IL-1β and cells death. The effect of these all could result in induction of necrotic core formation, which in turn may lead to overwhelming plaque destabilization leading to plaque rupture. Indeed, we have observed that NLRP3-inflammasome activation in VSMCs is associated with plaque rupture in human carotid artery disease.

IL-1 isoform can act extracellularly in an autocrine or paracrine manner [9], while IL-1β induce its own gene expression in an amplification loop manner called autoinduction [10,11]. Secondary necrosis of apoptotic VSMCs promotes the release of both IL-1α and IL-1β, which induces the surrounding viable VSMCs to produce proinflammatory cytokines, thus causing a chronic inflammation associated with atherosclerosis [35]. In the present study, we could demonstrate that IL-1β triggers VSMCs phenotypic switch and transdifferentiation to macrophages-like cells whose effect was amplified in the presence of oxLDL. Remarkably, IL-1β treatment increased profoundly the lipids accumulation and VSMCs foam cells formation highlighting the critical role of IL-1β in atherosclerosis progression as well as VSMCs foam cells formation, with consequences for atherosclerotic plaque stability. Interestingly, ZVAD-FMK a cell-permeable pan-caspase inhibitor partly abrogated VSMCs phenotypic switch as well as foam cells formation. These findings clearly demonstrate that caspase inhibition might be a way to preserve VSMCs contractile phenotype. More interestingly, we could demonstrate the role of NLRP3 inflammasome activation in VSMC phenotypic triggered by OxLDL or OxLDL-activated monocytes or IL-1β using MCC950, a potent highly specific small molecule inhibitor of both canonical and noncanonical activation of NLRP3 inflammasome leading to the reduction of IL-1β production [22]. 

Current widely used anti-atherosclerosis therapies modulate only the factors associated with the development of the disease while growing evidence supports a role for inflammation in atherosclerosis. Colchicine is a small molecule, a natural product derived from the autumn crocus plant which has been used to treat chronic auto-inflammatory conditions [36] as well as pericarditis, stable coronary artery disease, and postpericardiotomy syndrome [37]. Colchicine interferes with the assembly of microtubules and in this way, it impedes the assembly of the multiple components that comprise inflammasomes and thus colchicine blocks inflammasome assembly and thus IL-1β production [38]. Colchicine is currently under extensive evaluation for safety and efficacy in large randomized controlled trials. The COLCOT (Colchicine Cardiovascular Outcomes Trial) and LoDoCo2 (Low Dose Colchicine2) trial both demonstrated that low-dose colchicine is efficient in preventing major adverse cardiovascular events [23]. Among the ongoing trials it is worth mentioning the COLPOT trial in patients with recent acute coronary syndromes, the CLEAR-SYNERGY (OASIS-9) trial in patients with STEMI undergoing percutaneous coronary intervention (PCI) or the CONVINCE trial which will determine the long-term tolerability and efficacy of low-dose colchicine for secondary prevention in patients with CAD [39]. However, there is a need for mechanistic studies explaining the athero-protective effects of colchicine and particularly whether colchicine could affect VSMC phenotypic switch and subsequently plaques destabilization. Our present finding shows the direct inhibitory effect of colchicine on oxLDL-induced VSMC phenotypic switch which could at least partly explain the atheroprotective effect of colchicine in preventing major adverse cardiovascular events [23]. 

The finding of this study goes beyond the simple understanding of the pathogenesis of atherogenesis, since it provides a new mechanistic insight of therapeutic strategies preventing plaque destabilization and major adverse cardiovascular events. Taken all together our data implies that NLRP3 inflammasome activation is a critical mechanism involved in VSMCs phenotypic switch with possible implications in human atherosclerotic plaque destabilization. 

## 4. Materials and Methods

### 4.1. Animals

Eight to twelve-week-old C57BL/6 mice were used for VSMCs or monocytes isolation. Eleven-week-old male Apoe^−/−^ or Apoe^−/−^ Myh11-CreERT2, ROSA26 STOP-flox eYFP^+/+^ mice were fed a NCD (4.6% fat, 21.1% protein, 4.5% fiber, 6.4% ash, Special Diets Services, UK) for 16 weeks (early atherogenesis) [40] or a HCD for 11 weeks (20.1% fat, 1.25% cholesterol, Research Diets, Inc., New Brunswick, New Jersey, United States) to promote advanced atherogenesis [41]. To facilitate VSMCs lineage tracing, injection of tamoxifen was used to induce Cre recombinase activation in male Apoe^−/−^ Myh11-CreERT2, ROSA26 STOP-flox eYFP^+/+^ mice [42]. A series of ten intraperitoneal 1 mg tamoxifen (Sigma) injections from 9 to 11 weeks of age, for a total of 10 mg of tamoxifen per mouse, and an average bodyweight of 25 g for the 2 weeks running up to the start of the high cholesterol diet was performed [15]. Whole blood was collected and serum triglycerides, total cholesterol, low-density lipoprotein-cholesterol (LDL-C) were measured. Animals were sacrificed by exsanguination after anesthesia with 4% isoflurane. Experimental protocols and procedures were reviewed and approved by the Institutional Animal Care and Use Committee of the Geneva University School of Medicine. Animal care and experimental procedures were carried out in accordance with the guidelines of the Institutional Animal Care and Use Committee of the Geneva University School of Medicine. All procedures conform to the guidelines from Directive 2010/63/EU of the European Parliament on the protection of animals used for scientific purposes or the NIH Guide for the Care and Use of Laboratory Animals. 

### 4.2. Human Samples

Specimens of internal carotid plaques of a previously published cohort study [43] from symptomatic patients with CAD and a first episode of ipsilateral ischemic stroke (ipsilateral focal neurological deficit of acute onset lasting >24 h), as well as of asymptomatic patients (no history of ischemic symptoms) undergoing endarterectomy for severe carotid stenosis were used for immunofluorescent analysis. Carotid endarterectomy (CEA) was performed due to extra cranial high-grade internal carotid stenosis (>70% luminal narrowing) in symptomatic and asymptomatic patients. US Doppler echography and angiographic confirmation using the criteria of the North American Symptomatic Carotid Endarterectomy Trial (NASCET) [44] was applied to determine the degree of luminal narrowing. The indication for CEA for asymptomatic patients was based on the recommendations of Asymptomatic Carotid Surgery Trial (ACST) [45] while for symptomatic patients, CEA indication followed the recommendations of the European Carotid Surgery Trial (ECST) [46] and the North American Symptomatic Carotid Endarterectomy Trial (NASCET) [46]. After surgical excision, the internal carotid plaque specimens were cut perpendicular to the long axis through the point of maximum stenosis to obtain the atherosclerotic plaque upstream to the blood flow. The upstream internal carotid plaque specimens from symptomatic and asymptomatic patients were embedded in optimal cutting temperature (OCT) compound. The study was approved by the Medical Ethics Committee of San Martino Hospital in Genoa (Italy) and conducted in compliance with the Declaration of Helsinki after participants provided written informed consent.

### 4.3. Cells Isolation

VSMCs isolation from the aorta of 8–12-weeks old C57BL/6 mice was successfully established in the laboratory. Briefly, after intracardial perfusion, the aorta is surgically excised. The aortic arch is separated from the thoracic part of the aorta. The aorta adventitia was carefully excised by sharp surgical dissection in a clearly defined plan, to leave a naked media over the length of the aortal segment. The intima was scrapped softly to eliminate endothelial cells. The obtained arch is digested for 40 min—1 h at 37 °C in DMEM containing Collagenase P, Dispase and DnaseI. VSMCs were isolated from via digestion at 37 °C in DMEM containing Collagenase P, dispase and DnaseI. VSMC phenotype was confirmed by flow cytometry analysis for smooth muscle α-actin and Myh11 positive expression and negative expression of CD31 (endothelia cell marker) and CD90 (fibroblasts cell marker). These cells were cultured at a density of 3 × 104 cells/cm^2^ using SmBMTM Basal Medium (CC-3181, Lonza) and SmGMTM-2 SingleQuotsTM supplements (CC-4149, Lonza) required for growth of VSMC for 3 weeks. The medium was renewed every 3 days. Briefly, the iliac, tibiae, and femur marrow cells were obtained from 8–12-weeks old C57BL/6 mice via flushing with cold PBS using a 22-gauge needle and passing the cell suspension through a 40-μm cell strainer (BD Biosciences, MD, USA). Mononuclear cells from blood were obtained after centrifugation by density gradient sedimentation using Histopaque (Sigma). Erythrocytes were lysed and nucleated cells were washed twice, counted, and suspended in PBS. Monocytes were isolated using mouse Monocyte Isolation Kit (BM) (Miltenyi Biotec, 130-100-629) according to the manufacturer instructions under sterile conditions. Dead cells and doublets were excluded based on exclusion dye or forward scatter profiles, respectively. Monocytes cell purity (>95%) and phenotype were confirmed by flow cytometry using Anti-Ly-6C-FITC, mouse (Miltenyi Biotec, 130-102-295) CD11b-VioBlue, (Miltenyi Biotec, 130-113-810).

### 4.4. Flow Cytometry Analysis of Vascular Smooth Muscle Cells Phenotypic Switch

Quantification of VSMC transdifferentiation was performed using VSMC in passage 1. In vitro VSMC were stimulated with either 40 ng/mL oxLDL (Thermo fisher), Z-VAD-FMK 10 μM (InvivoGen) or 100 ng/mL Colchicine (Sigma-Aldrich), or 10 ng/mL of IL-1β for 7 days or co-cultured with monocytes derived from male C57Bl/6 mice using Transwell Cell Culture Inserts for 7 days. VSMCs were co-cultured with monocytes or monocytes activated with oxLDL upon direct supplementation of 40 ng/mL oxLDL (Thermo Fisher) to well cell culture inserts (pore size 0.02 μm) in the trans well plates for 7 days. The direct supplementation of oxLDL with a known diameter size of more than 20 nm [47] ensured monocytes restricted oxLDL activation since oxLDL was retained in the trans well inserts with a pore size of 0.02 μm. Quantification of VSMC transdifferentiation was performed via flow cytometry analysis of anti-mouse CD68 PerCP/Cy5.5 (Biolegnd), anti-mouse MAC2 PE/Cy7 (Biolegnd), anti-mouse F4/80 Brilliant Violet 650™, α-SMA Alexa Fluor 488, SM22α^+^ Alexa Fluor 700 after excluding dead cells via LIVE/DEAD Fixable Near-IR Dead Cell Dye staining (Thermo fisher). Samples were acquired in Gallios flow cytometer (Beckman Coulter) and analyzed using FlowJo software (TreeStar, Version 10.0.8r1, Ashland, OR, USA).

### 4.5. Quantitative Real-Time PCR

Total mRNA was prepared by Trizol^®^ (Thermofischer), according to the provider protocol. Reverse transcription was performed using the ImProm-II Reverse Transcription System (Promega, Madison, WI, USA) according to the manufacturer’s instructions. Real-time PCR (StepOne Plus, Applied Biosystems, Waltham, MA, USA) was performed with the SensiFast (LabGene). Real-time duplex qPCR analysis was conducted. The levels of mRNA expression were normalized against the expression of a housekeeping gene (hprt) and analyzed using the comparative ΔCT method. Probes were purchased from Applied Biosystems. All measurements were conducted in triplicate.

### 4.6. Immunofluorescent Staining and Quantification

VSMCs stimulated with oxLDL or co-cultured with monocytes or oxLDL-activated monocytes were cultured in 6-well chamber slides for 24 h at 37 °C with 5% CO_2_. The cells were fixed with 4% paraformaldehyde for 30 min at room temperature (RT), permeabilized for 30 min with PBS plus 0.01% Triton X-100 for 30 min, and stained with Phalloidin (Abcam) for 1 h at RT followed by three washing steps for 10 min and counterstained with ASC (Cell Signaling). Confocal microscopy was performed with a confocal LSM 800 Airyscan (Zeiss). Internal carotid plaque specimens from symptomatic and asymptomatic patients, and the aortic roots of male Apoe^−/−^ Myh11-CreERT2, ROSA26 STOP-flox eYFP^+/+^ mice on NCD and HCD were embedded in OCT serially cut into 5-μm sections. Cryosections were fixed in 1% paraformaldehyde and then washed with 1xPBS and incubated with blocking solution, consisting of 5% BSA in PBS for 30 min, then permeabilized with Triton X-100 0.1%. Endarterectomy specimens were stained with primary anti-Myh11 (Thermo Fischer) and CD68, cleaved caspase 1 or Il-1β antibody (Cell Signaling) in blocking solution. After washing, the samples were incubated with secondary antibody and mounted with ProLong Glass Antifade Mountant (Thermo Fischer). Immunofluorescent images will be acquired with Axioscan Z1 microscope, analyzed, and quantified with QuPath software platform for whole slide image analysis. The extent of VSMC phenotypic switch/NLRP3 inflammasome activation was corelated with the risk of CAD events in human atherosclerosis using the two groups of CAD patients (symptomatic versus asymptomatic). Aortic roots cryosections of Apoe^−/−^ or Apoe^−/−^ Myh11-CreERT2, ROSA26 STOP-flox eYFP^+/+^ mice fed NCD or HCD were stained with primary rabbit anti-CD68 (BioRad), cleaved caspase 1 (Cell Signaling), NLRP3 (Cell Signaling), α-SMA (Abcam), or IL-1β (Cell Signaling) antibody in blocking solution. After washing, the samples were incubated with the following secondary antibody Alexa 647 anti-rabbit (Thermo Fischer) and DyLight 405 and mounted with ProLong Glass Antifade Mountant (Thermo Fischer). Immunofluorescent images were acquired with Axioscan Z1 microscopy and analyzed and quantified with QuPath software platform for whole slide image analysis. 

### 4.7. Caspase-1 Activity Assay and Pyroptosis/Caspase-1 Assay

Caspase 1 activity was measured with a caspase-1 colorimetric assay (R&D Systems, Minneapolis, MN, USA) according to the manufacturer’s protocol. In brief, 50 µL containing 100 µg of VSMCs protein extract was mixed with 50 μL of 2X Reaction Buffer 1 and 5 μL of a caspase-1 colorimetric substrate and incubated for 2 hours at 37 °C. The caspase 1 activity in the samples was quantified with a microplate reader using a wavelength of 405 nm. Data represent the absorbance of the samples. For pyroptosis/caspase-1 assay, caspase-1 activity was assessed in whole VSMC cells in vitro treated with oxLDL or co-cultured with monocytes or oxLDL-activated monocytes as previously described, using FAM-YVAD-FMK Pyroptosis/Caspase-1 Assay, Green (ImmunoChemistry Technologie), according to the manufacturer’s protocol. The activity of the caspase-1 enzyme inside the cells was quantified using a cell-permeant FLICA retaining the green, fluorescent signal within the cell, with no interference from pro-caspases or inactive forms of the enzymes. To access pyroptosis, after labelling with FLICA, VSMCs were counter-stained with the red live/dead stains propidium iodide (ImmunoChemistry Technologie) and 7-AAD (ImmunoChemistry Technologie) and the fluorescence signal was quantified via flow cytometer. Samples were acquired in Gallios flow cytometer (Beckman Coulter) and analyzed using FlowJo software (TreeStar, Version 10.0.8r1).

### 4.8. IL-1β ELISA

To measure IL-1β secretion specifically in VSMSs upon oxLDL or co-culture with monocytes or oxLDL activated monocytes as previously described, the transwell inserts were removed and the VSMC in the well plates were supplemented with fresh medium, which was collected after 3 days and IL-1β secreted by VSMCs was determined using a mouse IL-1β ELISA kit (Cloud Clone Corp., Houston, TX, USA) according to the manufacturer’s protocol.

### 4.9. Statistical Analysis

Statistical analysis was performed using a GraphPad Software, Inc., La Jolla, CA, USA. All data sets were tested for normal distribution with normality tests before proceeding with parametric or non-parametric analysis. Grubb’s test was performed in order to exclude spurious outliers. Statistical significance was tested using unpaired *t*-test, one-way analysis of variance (ANOVA) with Tukey post-test and two-way ANOVA with Bonferroni post-test for data sets with normal distributions. Statistical significance was tested with Mann–Whitney test and one-way ANOVA with Dunn’s post-test for data sets without a normal distribution. Data are presented as mean ± SEM. Differences were significant when the two-sided *p*-value was lower than 0.05.

## Data Availability

Data is contained within the article or Supplementary Material.

## Supplementary Materials

The following are available online at https://www.mdpi.com/article/10.3390/ijms23010340/s1.
