# Supplementary material for: NLRP3 Inflammasome Activation Controls Vascular Smooth Muscle Cells Phenotypic Switch in Atherosclerosis
Source: Int J Mol Sci. 2021 Dec 29;23(1):340. doi: 10.3390/ijms23010340 (PMC8745068; doi:10.3390/ijms23010340)
Supplement: Supplementary file 1 [file ijms-23-00340-s001.zip › ijms-1434164-supplementary.pdf]

Supplementary Materials: The following are available online at www.mdpi.com/xxx/s1,

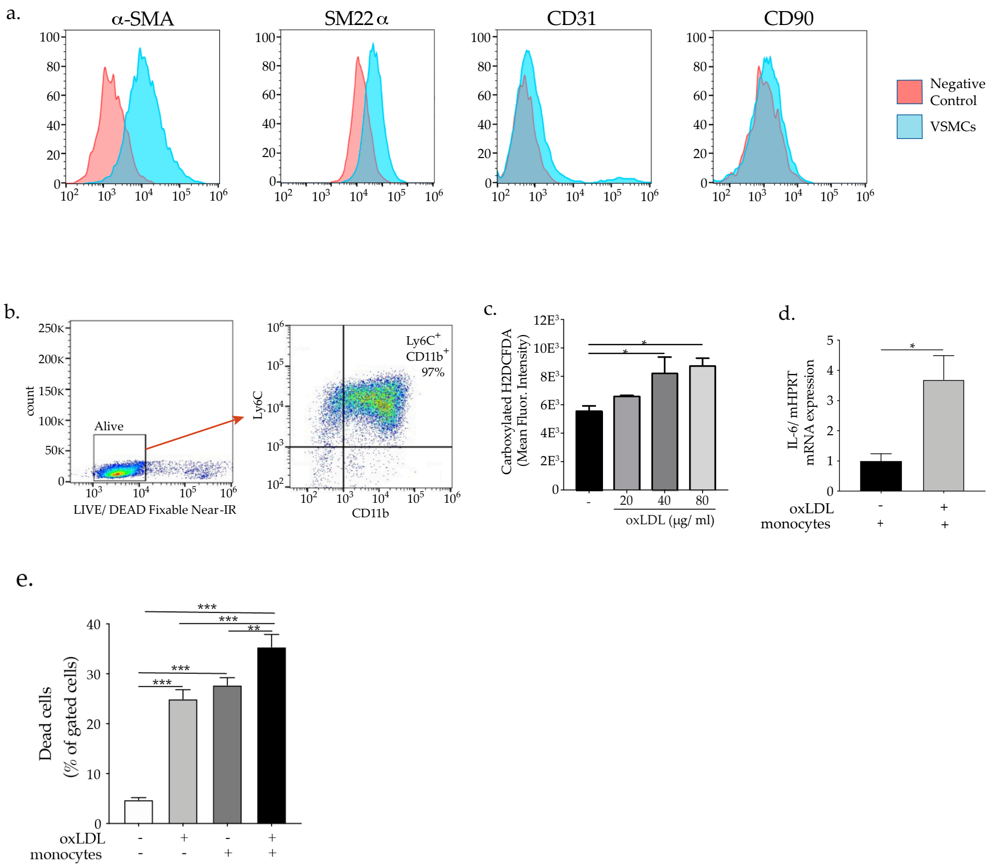

Figure S1: (a) Histograms represent the express of VSMC-specific markers α-SMA, SM22α, and negative expression of endothelial cells marker CD31 and fibroblast marker CD90 in the isolated mouse VSMC. (b) Mouse monocytes purity check-up. (c) Dose dependent ROS production in oxLDL-activated monocytes. Graph bars represent the mean ± SEM of mean fluorescent intensity of carboxylated H2DCFDA mouse monocytes with n = 6/group and *p < 0.05, One-way ANOVA. (d) Graph bars represent the mean ± SEM of mRNA expression of IL-6 in monocytes or oxLDL-activeted monocytes, as indicated, with n = 6/group and *p < 0.05, unpaired t-test. (e) Graph bars represent the mean ± SEM of Propidium Iodide/7-AAD expressing VSMCs as a percentage of gated VSMCs as indicated, with n = 6/group, **p < 0.01, ***p < 0.001, One-way ANOVA.

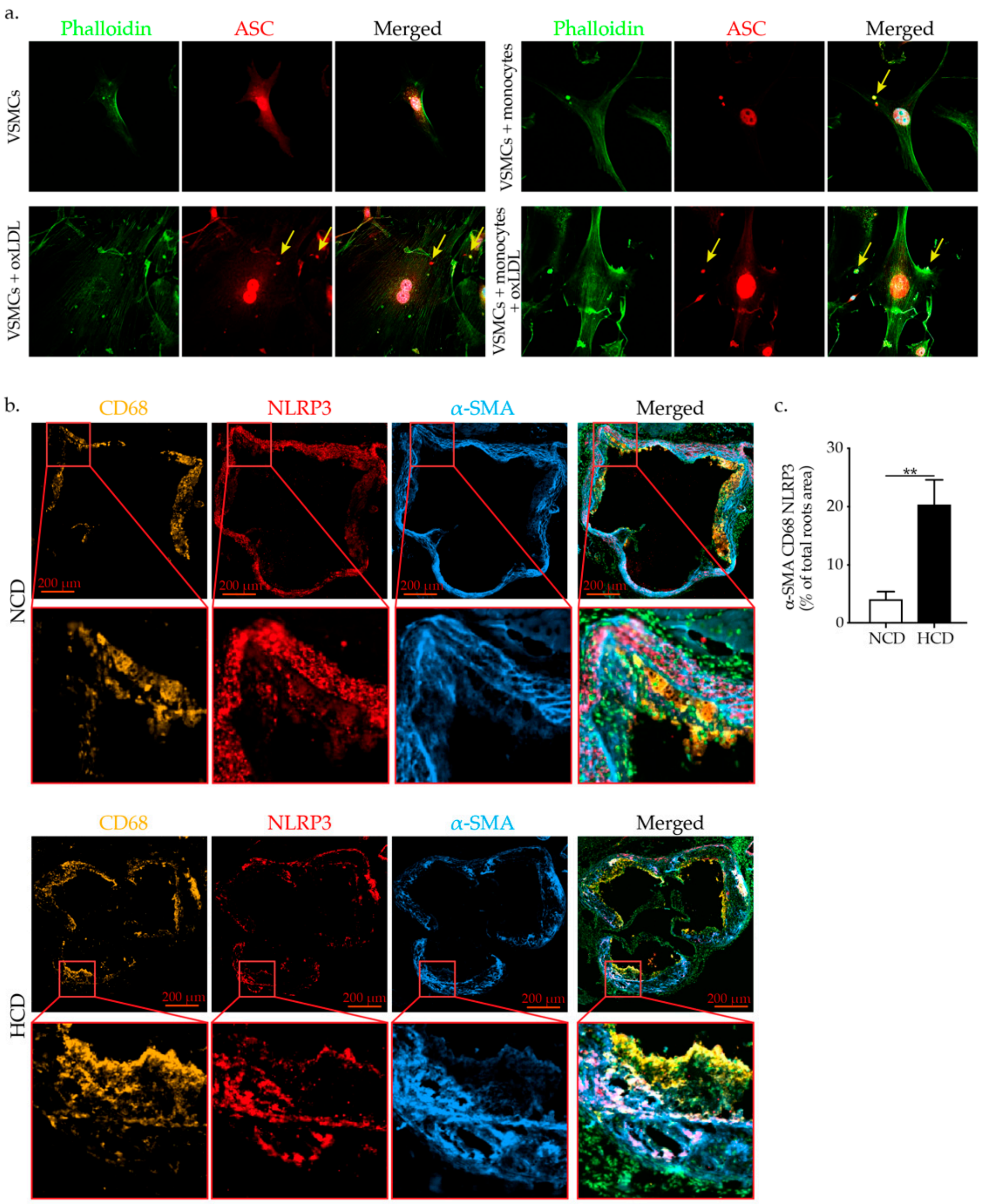

Figure S2: (a) NLRP3 Inflammasome Activation in VSMCs. (a) ASC speck formation (yellow arrow) by confocal microscopy (LSM 800 Airyscan) and immunofluorescence imaging in VSMCs treated with oxLDLs or co-cultured with monocytes or oxLDL-activated monocytes. (b) CD68, NLRP3, α-SMA in the aortic roots of Apoe$^{-/-}$ mice fed NCD or HCD. (c) Graph bars show the mean ± SEM of CD68, NLRP3, α-SMA co-expression in the aortic roots plaques of Apoe$^{-/-}$ mice fed NCD or HCD, n = 8/group and **$p < 0.01$, unpaired t-test.
